# Feasibility of ambulatory surgery for anal fistula with LIFT procedure

**DOI:** 10.1186/s12876-019-0997-x

**Published:** 2019-05-30

**Authors:** Jian-ming Qiu, Guan-gen Yang, Hong-tao Wang, Chao Fu, Dong Wang, Tingting Mei

**Affiliations:** The Coloproctology Department, The Third Hospital of Hangzhou, 38 West Lake Ave, Hangzhou, China

**Keywords:** Ambulatory surgery, Anal fistula, LIFT

## Abstract

**Background:**

Ambulatory surgery maintains the advantages of a more rapid return to work and overall reduced hospital costs. The specific impact of ambulatory surgery for anal fistula using the LIFT procedure (ligation of the intersphincteric fistula tract) is presented.

**Methods:**

A total of 218 consecutive patients with anal fistula who underwent ambulatory LIFT surgery were retrospectively compared with 386 cases managed as in-patients. Patient demographics, comorbidities, postoperative morbidity and pain as well as readmission rates within 30 days and satisfaction ratings were compared between the two groups.

**Results:**

When compared with patients undergoing in-patient surgery, those in the ambulatory group were younger with a better level of education (*P* < 0.05). Ambulatory cases returned to work after shorter postoperative periods (*P* < 0.01) but experienced more frequent postoperative external hemorrhoidal thrombosis and more reported postoperative pain (*P* < 0.05). There were no differences in the overall rate of complications or readmissions between the two groups. Ambulatory patients reported higher satisfaction ratings than in-patients (*P* < 0.05).

**Conclusions:**

The LIFT procedure for anal fistula can be safely performed in the ambulatory setting resulting in an acceptable level of satisfaction and a more rapid return to work when compared with in-patient fistula management.

## Background

Benign proctological problems including haemorrhoids, anal fissure, perianal abscess and cryptogenic anal fistula are common [1] with a limited assessment of the value of ambulatory surgery (completion of surgery and discharge within a 24-h period). It is anticipated that an ambulatory approach towards selected benign anorectal disease will accrue the benefits of ambulatory surgery for other conditions, namely a more rapid return to work and an overall reduced hospital and social cost [2–4]. The limited data shows such advantage in a range of benign anorectal conditions including fissure, haemorrhoids, fistula, pilonidal sinus, anal condylomata and perianal abscesses [5]. To date, there are no reports specifically comparing the impact of ambulatory surgery on particular types of more minimally invasive fistula surgery such as the Ligation of the Intersphincteric Fistula Tract (LIFT) procedure [6, 7]. This single institution study retrospectively assesses the initial safety, perioperative pain, postoperative complications and return to work of ambulatory LIFT surgery comparing it with in-patient fistula management.

## Methods

### Data collection and follow-up

Approval for the conduct of this retrospective data was provided by the local hospital Ethics Committee with all patients providing informed consent for their procedures. A total of 604 consecutive patients with anal fistula underwent LIFT surgery between January 2012 and December 2017 at the Proctology Department of The Third Hospital of HangZhou, China, (a dedicated State-run tertiary referral center for anorectal diseases). Of the total cohort, there were 218 patients in the ambulatory surgery group (Group 1) and 386 in the in-patient surgery group (Group 2). The LIFT procedure which has been previously described [8] was performed in the standard manner by surgeons trained in the technique. Patients who’s up to the following criteria were enrolled for this study:1). Patients having no previous anal fistula operations, inflammatory bowel diseases (IBD),tuberculosis or STD.2). Patients with an ASA I ~III levels (ASA, American Society of Anesthesiology).3). Those who were not on anticoagulant therapy 1 week prior to the procedure.4). An informed willingness to choose procedure on an ambulatory or inpatient setting based on the evaluation and discussion with surgeons.5). Patients having a responsible adult family member for postoperative care after ambulatory surgery.6). Willing to join the follow up program after discharge. The decision for ambulatory discharge (as defined) was based on the conscious state of the patient postoperatively and the absence of any bleeding from the wound. Patient demographics, comorbidities, operative details and perioperative, and postoperative data were collated along with the results of a satisfaction questionnaire. All patients were invited to join the Wechat cell phone communication App as a forum to report any adverse events following hospital discharge. Follow-up was routinely undertaken at one and 3 postoperative weeks and completed until there was either evidence of fistula healing or the passage of 3 postoperative months. Patients were asked to rate their satisfaction on a simple scale (low, medium or high) with the pain level graded at their first follow-up visit using a Visual Analogue Scale (VAS). Results were recorded by 2 outpatient nurses blinded to each other’ s findings and then averaged.

### Statistical analysis

All patient data were recorded on a dedicated database with statistical analysis performed using the SPSS v.11.5 software (SPSS Inc.,Chicago, USA). Parametric data were presented as means ± standard deviations (SD) and compared using the Student’s *t*-test. Nonparametric data were presented as medians (+ interquartile range) and compared with the Mann-Whitney U-test. Nominal data were compared with the χ2 test or Fisher’s exact method where appropriate with *P* values for two-sided significance < 0.05 considered significant.

## Results

Comparative demographic data are shown in Table 1. The median age of the 218 Group 1 fistula patients was 33 years (range 17–64 years) comprising 168 (77%) males and 50 (23%) females. The mean overall BMI in Group 1 was 27.5 (±5.3) with 47.7% of patients having a smoking history within a year before their surgery. Of Group 1 cases, 45% of the patients had a background of high education (either University or College degree). By comparison the median age of the 386 Group 2 patients was higher (46 years; range **Q write the range here**: *P* = 0.035) and these patients had a lower education standard (*P* = 0.042). No differences were noted in the individual comorbidities (diabetes, hypertension and coronary artery disease) between the two groups.

**Table 1:** Demographics of enrolled patients with anal fistulae (Groups 1 and 2)

Demographics	Ambulatory Surgery (*n* = 218) Group 1	Inpatient Surgery (*n* = 386) Group 2	P value
Median age (yrs)	33	46	0.035
Male/female	168/50	277/109	0.072
Mean BMI	27.5 ± 5.3	28.1 ± 4.8	0.827
Smoking	117	220	0.070
ASA level			0.804
I	199	358	
II	16	24	
III	3	4	
High education			0.042
Yes	98	114	
No	120	272	
Diabetes			0.622
Yes	31	44	
No	187	342	
Hypertension			0.752
Yes	50	88	
No	168	298	
Coronary heart disease			0.187
Yes	7	22	
No	211	364	
Fistula type			0.611
Simple	141	238	
Complex	77	148	

Table 2 shows the operative data for the two main groups. Even though all the patients underwent a classical LIFT procedure there were a range of anaesthetic options utilized with significantly more general anaesthesia used in Group 2 cases and more local and caudal anaesthesia used in Group 1 patients (*P* < 0.001). No differences were noted between the two main groups either in terms of operating time or estimated blood loss.

**Table 2:** Peri-operative parameters in the anal fistula cohort (Group 1 vs. Group 2)

Parameters	Ambulatory surgery Group 1 (%)	Inpatient surgery Group 2 (%)
Operative time (min)	38 ± 11	47 ± 19
Anesthesia approach*		
General	20 (9.2)	251 (65)
Spinal	67 (30.7)	88 (22.8)
Caudal	29 (13.3)	7 (1.8)
Local	102 (46.8)	40 (10.4)
Blood loss (mL)	17 ± 8	22 ± 10

^*^ Overall *P* value < 0.001

Overall, there was no difference noted between the groups in the incidence of postoperative complications (11.0% Group 1 vs. 7.8% Group 2, respectively) which included postoperative haemorrhage, urinary retention, thrombosed external piles, perianal sepsis and delayed wound healing (Table 3). Specifically, of those with complications more cases in Group 1 presented with a postoperative external haemorrhoidal thrombosis (*P* < 0.05).

**Table 3:** Summary of postoperative complications

Complications	Ambulatory surgery (Group 1) *n* = 218	Inpatient surgery (Group 2) *n* = 386
Haemorrhage	3	7
Urinary Retention	1	3
Thrombosed hemorrhoids^*^	11	2
Perianal sepsis	0	1
Delayed wound healing	9	17
Anal stenosis	4	5
Faecal incontinence	–	–
Recurrence	9	14

^*^P < 0.05

Readmissions within 30 days were due to secondary haemorrhage, perianal sepsis or delayed wound healing with no significant differences noted between Groups 1 and 2 (4.59% *vs*. 5.44%, respectively). Group 2 patients reported a better VAS at the first postoperative visit when compared with Group 1 cases (3.2 ± 1.*4 vs.* 6.8 ± 2.5, respectively; *P* < 0.05). with a significant earlier return to work in Group 1 patients when compared with Group 2 cases (36 ± 15 h *vs.* 120 ± 44 h, respectively; *P* < 0.01). Satisfaction was reported as high more often in Group 1 cases when compared with Group 2 patients (92.7% *vs.* 78%, respectively; *P* < 0.05) with no differences between groups in those reporting low satisfaction (2.7% vs. 4.4%, respectively).

## Discussion

Countries around the world are confronted with rising health care costs where there is a variety of options designed to reduce medical expenditure, one of which is an increase in ambulatory surgery [9]. Although ambulatory anal fistula surgery is common in developed countries such as the United States, developing countries (which in this respect can include China) do not generally use the ambulatory approach as part of standard fistula practice. As far as we are aware, this is the first report from China which addresses the impact of ambulatory surgery particularly when the LIFT procedure is used.

Our retrospective study over 5 years in a single institution has shown that ambulatory LIFT is feasible resulting in a more rapid return to work and superior patient satisfaction. It is accepted that in this study patients from both groups (ambulatory *vs.* in-patient) do not have strictly comparable demographics with ambulatory cases on average being younger and more educated. These patients generally cannot afford the time incurred with in-patient treatment and are more likely to have learned from internet searches or heard of the possibility and potential advantages of ambulatory care.

The rise of ambulatory surgery is related to the emergence of lower-risk and minimally invasive procedures. In this instance, the sphincter preserving LIFT approach is more suited to the day care setting (even in complex cases), although this decision will still be selective and case-dependent [10]. General anaesthesia was less commonly used in ambulatory cases where in the day case setting at our hospital we have limited resuscitation resources. Most of the ambulatory surgery was able to be performed under local or caudal anaesthesia, an effect which will impact total costs. This matter is complicated, however, if planned probing of the fistula is likely to change the operative decision away from LIFT where such cases will typically be commenced under general anaesthesia.

Even though the same analgesia was prescribed for all the patients, the ambulatory group reported worse early postoperative pain. This most likely reflects concerns that these patients have in the immediate postoperative period that they will have ready access to review by their surgeon and to strong analgesia. Overall, the postoperative complications in the two groups were similar and comparable with previous reports in the literature [11] although specifically there were more cases in the ambulatory patients of thrombosed external piles. Ambulatory patients reported higher satisfaction rates most likely because of the appeal .of a more rapid return to work and reduced expenses incurred if the option of a short period of hospitalization was waived. In summary, LIFT surgery for anal fistula can be performed in a day case setting with safety and high patient satisfaction. In China this approach combining the surgeon and the anaesthetist as a team in the selection of patients for day case LIFT surgery will result in the optimal utilization of medical resources. Future studies are still needed to analyze the longer-term LIFT outcomes as well as the effect on quality of life with this selective approach.

## Conclusion

LIFT surgery for anal fistula can be performed in the ambulatory setting as safely and with higher satisfaction rates as with in-patient management, reducing costs and resulting in a more rapid return to work.

## Data Availability

Data can be provided by the corresponding author upon request.

## Abbreviation

LIFT: Ligation of intersphincteric fistula tract
